# Mitral Regurgitation in Contemporary Practice: From Diagnosis to Management

**DOI:** 10.31083/RCM49149

**Published:** 2026-08-05

**Authors:** Carlo Gaspardone, Paolo Costa, Giuseppe Barone, Rosaria Sofia, Riccardo Mager, Francesca Braccischi, Riccardo Mazza, Marianna Adamo, Francesco Maisano, Cosmo Godino, Marco Metra, Gabriele Fragasso

**Affiliations:** ^1^Interventional Cardiology Unit, IRCCS San Raffaele Scientific Institute, 20132 Milan, Italy; ^2^Institute of Cardiology, ASST Spedali Civili, University of Brescia, 25123 Brescia, Italy; ^3^Vita-Salute San Raffaele University, IRCCS San Raffaele Scientific Institute, 20132 Milan, Italy; ^4^Department of Cardiac Surgery, Heart Valve Center, IRCCS San Raffaele Scientific Institute, 20132 Milan, Italy; ^5^Clinical Cardiology Unit, IRCCS San Raffaele Scientific Institute, 20132 Milan, Italy

**Keywords:** mitral valve insufficiency, heart valve diseases, echocardiography, heart failure, cardiac surgical procedures, cardiac catheterization, disease management, natriuretic peptides

## Abstract

Mitral regurgitation (MR) is the most prevalent valvular heart disease in developed countries, affecting approximately 1 in 50 individuals in the general population and about 1 in 10 individuals older than 75 years. Despite its frequency, MR remains a complex clinical challenge due to its heterogeneous etiology, variable natural history, and evolving therapeutic landscape. The 2025 ESC/EACTS guidelines have introduced a paradigm shift, transitioning from a purely morphological classification to a mechanism-based framework that distinguishes primary, ventricular secondary, and atrial secondary MR. This review provides a comprehensive overview of contemporary MR management. We discuss the updated classification system and the associated implications for risk stratification, emphasizing how the underlying mechanism of MR influences prognosis and guides treatment selection. The diagnostics section addresses multimodality imaging strategies, detailing the complementary roles of echocardiography, cardiac magnetic resonance (CMR), and cardiac computed tomography (CCT) in assessing severity, characterizing anatomy, and planning interventions. The management section addresses optimization of medical therapy, surgical techniques (repair versus replacement, including conventional and minimally invasive approaches), and transcatheter interventions, with particular focus on mitral transcatheter edge-to-edge repair (M-TEER) and emerging transcatheter mitral valve replacement technologies. We propose an integrated, stepwise management algorithm that incorporates early risk markers, including natriuretic peptides, to guide the timing of intervention before irreversible myocardial damage occurs. The critical role of multidisciplinary heart valve teams (HVTs) in individualizing treatment decisions is highlighted throughout. Early recognition of MR and timely intervention are paramount to prevent progression to irreversible left ventricular dysfunction and heart failure. The mechanism-based classification enables more precise patient phenotyping and treatment tailoring. Transcatheter therapies have expanded therapeutic options for high-risk surgical patients, although careful patient selection remains essential. Natriuretic peptides have emerged as valuable biomarkers for identifying candidates who may benefit from earlier intervention. Contemporary MR management requires the integration of advanced imaging, mechanism-based classification, and collaborative decision-making within HVTs. As therapeutic options continue to expand, optimal outcomes depend on matching the right intervention to the right patient at the right time.

## 1. Introduction

Mitral regurgitation is the most common valvular heart disease in developed countries, with an estimated prevalence of 2–3% in the general population and over 10% among individuals older than 75 years [1]. When considering only patients with a hemodynamically significant disease (moderate-to-severe MR), the trend is similar, with prevalence increasing along with the age of the population investigated [2,3]. Beyond its prevalence, significant MR carries clear clinical weight: it is associated with higher mortality, progressive LV dysfunction, atrial fibrillation (AF), pulmonary hypertension, and right-sided HF. With the steady decline in coronary mortality over recent decades, MR has become one of the leading residual drivers of the HF burden, especially in older populations [4].

The natural history of MR is heterogeneous. Some patients remain asymptomatic for years, while others progress rapidly to dyspnea, atrial arrhythmias, or overt HF. Once symptoms emerge, or once ventricular dysfunction becomes evident, the prognosis significantly worsens unless the regurgitation is corrected [5]. Even moderate MR can accelerate disease progression in susceptible patients, indicating that it actively contributes to adverse outcomes rather than merely accompanying them [6,7].

The aim of this review is to offer cardiologists, interventional cardiologists, imaging specialists, cardiac surgeons, fellows, and medical students a practical overview of MR in current clinical practice, covering pathophysiology, natural history, diagnostic work-up, and therapeutic options. We also bring together the new 2025 ESC/EACTS mechanism-based classification, multimodality imaging, recent transcatheter advances, and emerging biomarkers within a management algorithm applicable to everyday practice, with the aim of changing the trajectory of a disease historically considered relentlessly progressive.

## 2. Classification, Pathophysiology, and Natural History

**Fig. 1.**
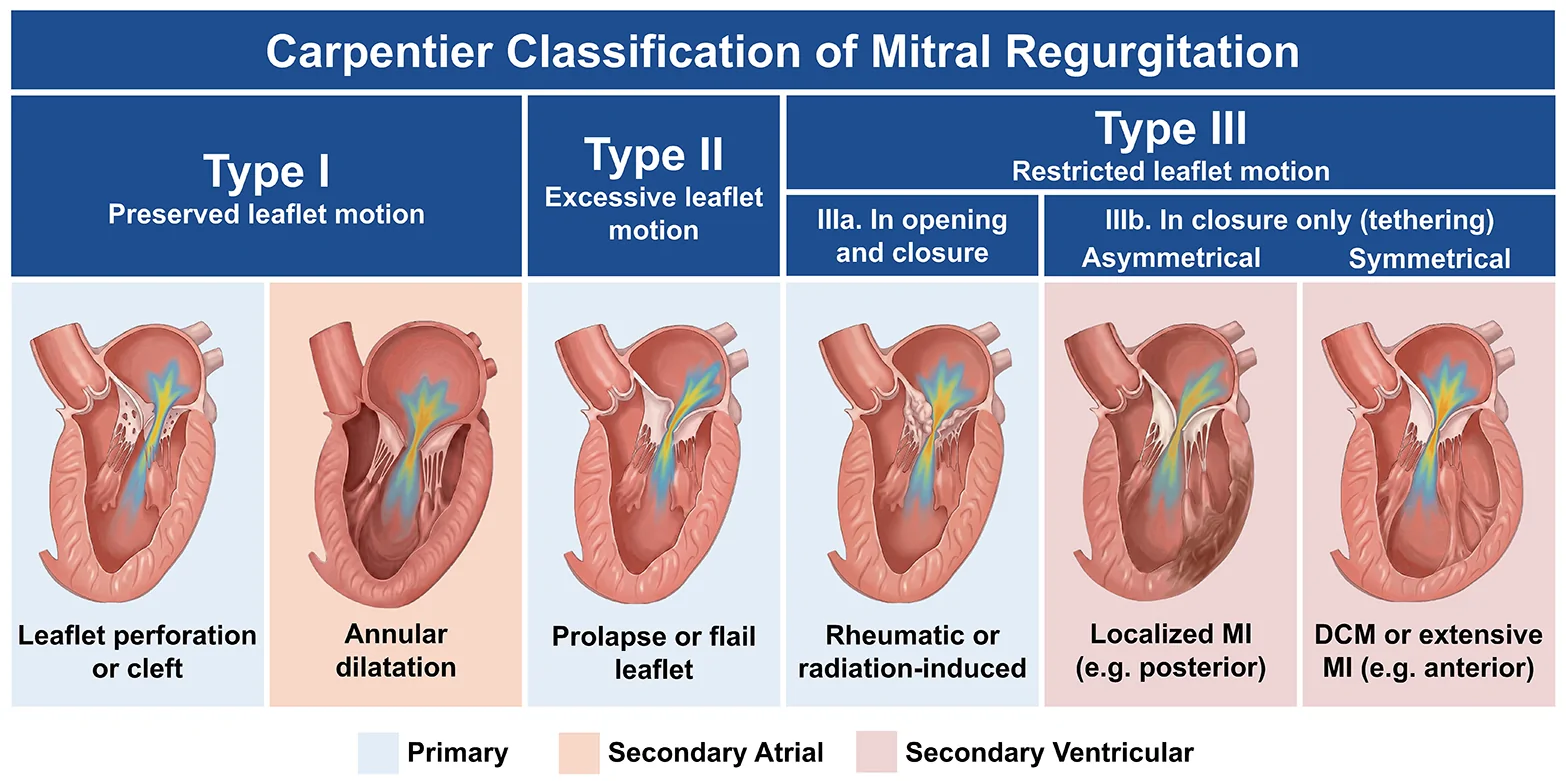
**Integration of Carpentier classification with mechanism-based classification of mitral regurgitation**. Caption: The Carpentier classification is based on leaflet motion patterns: Type I (preserved motion) can result from leaflet perforation in endocarditis, congenital cleft, or annular dilatation secondary to atrial enlargement in atrial fibrillation; Type II (excessive motion) includes mitral prolapse and flail leaflet; Type III (restricted motion) is subdivided into IIIa (restriction in both diastole and systole) as seen in rheumatic disease, mitral annular calcification, radiation-induced valvulopathy, etc., and IIIb (restriction in systole only due to leaflet tethering). Type IIIb manifests as asymmetrical tethering in localized myocardial infarction (MI) such as posterior MI, or symmetrical tethering in dilated cardiomyopathy (DCM) or extensive anterior MI with adverse ventricular remodeling. The contemporary mechanism-based classification overlays these anatomical patterns: Primary MR (blue), arising from intrinsic valve disease, predominantly corresponds to Type II, Type IIIa, and certain Type I etiologies; Atrial Secondary MR (orange) corresponds to Type I with annular dilatation while Ventricular Secondary MR (pink) corresponds to Type IIIb. This integrated framework guides therapeutic decision-making by linking anatomical substrate to underlying pathophysiological mechanism.

MR is now viewed as a spectrum of disorders rather than a single entity, reflecting the close interaction between valve leaflets, annulus, subvalvular apparatus, left ventricle, and left atrium. The 2025 ESC/EACTS guidelines have formally adopted this view, moving from the purely morphological Carpentier classification to a framework based on the underlying mechanism and the chamber of origin. Three main categories are now recognized: primary MR (PMR), ventricular secondary MR (ventricular SMR), and atrial secondary MR (atrial SMR) [8,9] (Fig. 1).

In primary, or degenerative, MR, the pathology lies in the valve itself, i.e., in one or more of the three main components of theMV apparatus (valve leaflets, chordae tendinae, and papillary muscles). Typical clinical scenarios include myxomatous degeneration, typified by Barlow’s disease, which produces redundant, thickened leaflets prone to prolapse or flail, or fibroelastic deficiency, which results in thin, fragile leaflets that often fail abruptly with chordal rupture [10]. Rheumatic involvement, radiation-induced injury, and infective endocarditis complete the etiological spectrum. The hemodynamic burden of primary MR is initially well tolerated: the low-impedance regurgitant orifice allows the ventricle to eject into the left atrium with minimal rise in systolic pressure. However, this initial compensation hides a progressive maladaptive process. Chronic volume overload drives left atrial enlargement, predisposes to AF, and gradually impairs ventricular contractile reserve. Consequently, in this context an ejection fraction (EF) of 60% may already signal early systolic dysfunction, since part of the stroke volume (SV) is ejected backwards into the atrium. In primary MR, MV surgery is therefore the treatment of choice once the criteria for intervention are met.

Ventricular secondary MR, by contrast, reflects pathology of the ventricle rather than the MV apparatus. Ischemic remodeling or dilated cardiomyopathy (DCM) distort ventricular geometry, displacing papillary muscles apically and laterally, and causing leaflet tethering and annulus dilation. The regurgitant orifice is typically crescent-shaped, dynamic, and apically displaced. Once established, MR worsens volume overload, which in turn promotes further ventricular dilatation and tethering, perpetuating the regurgitation. This type of MR is often divided into two subgroups: proportionate ventricular SMR, in which MR severity is proportionate to LV dilation (e.g., in a severely dilated LV with global dysfunction), and disproportional ventricular SMR, in which MR severity exceeds the degree of LV dilation (e.g., in case of regional wall abnormalities or ventricular dyssynchrony) [11]. This distinction is clinically relevant for guiding treatment selection. Typical clinical scenarios include HF with reduced ejection fraction (HFrEF), patients with ischemic heart disease or DCM. Prognosis is therefore dominated by the underlying cardiomyopathy, and observational data consistently show that even moderate ventricular SMR translates into excess mortality, recurrent hospitalizations, and faster progression to advanced HF, with a five-year survival often below 50% [6,12]. Treatment relies on a foundation of guideline-directed medical therapy (GDMT) for HF, with transcatheter interventions recommended when specific clinical and anatomical criteria are met.

The third entity, atrial secondary MR, has been codified only recently, but is now formally acknowledged in the 2025 guidelines [8,13]. In this case, atrial dilation leads to an enlarged and flattened mitral annulus, preventing normal leaflet coaptation. Patients often present with signs and symptoms of diastolic dysfunction, HF with preserved ejection fraction (HFpEF) or restrictive cardiomyopathies, obesity, and long-standing AF [14]. Regurgitation is frequently dynamic, worsening with exercise when atrial pressures rise. Recent data indicate that atrial SMR actively contributes to symptoms, arrhythmia progression, and higher hospitalization rates, rather than being a simple consequence of left atrial disease [15]. Therapy is most often based on treating the underlying comorbidities, e.g., HFpEF, obesity, and AF.

**Fig. 2.**
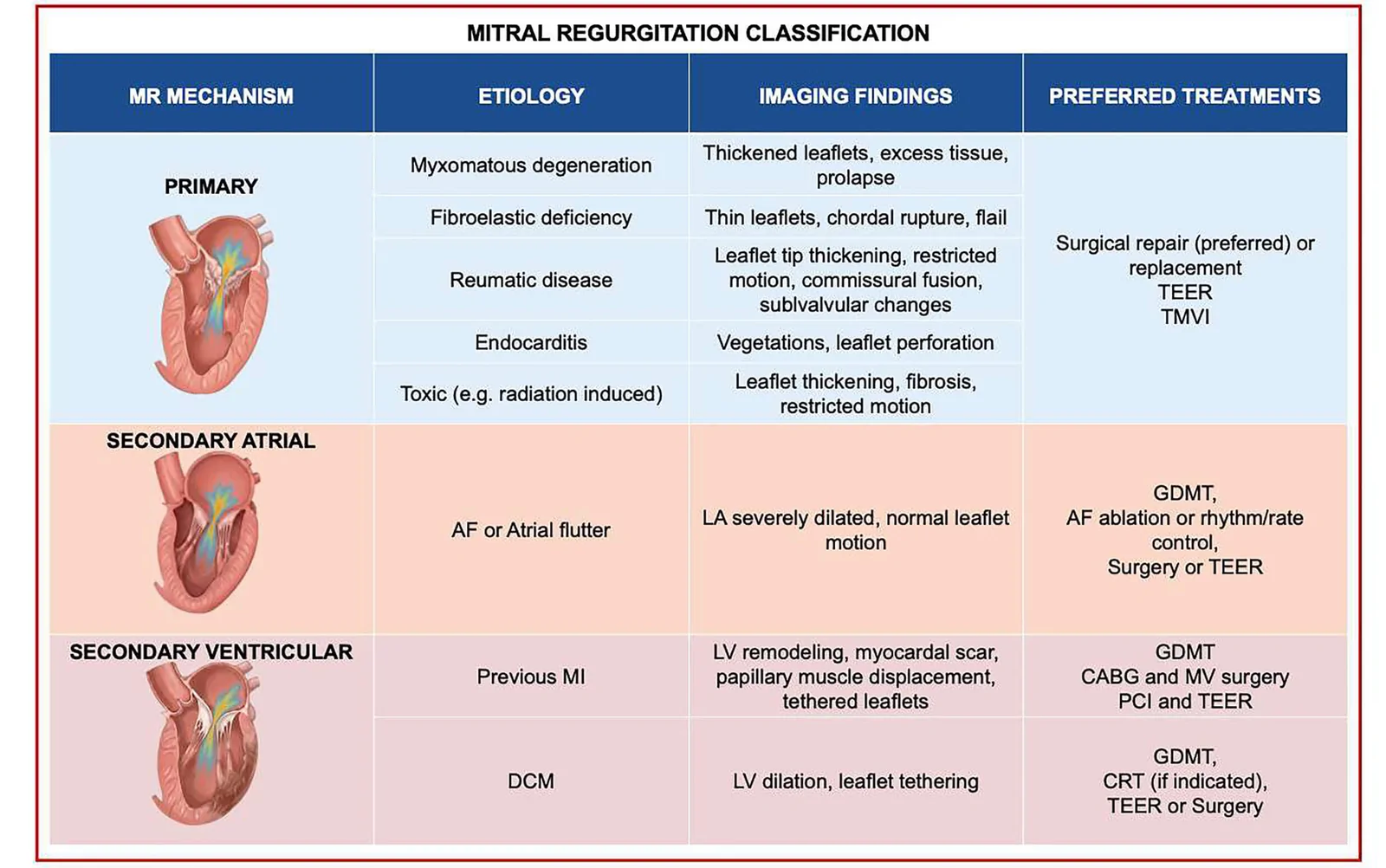
**Mechanism-based classification of mitral regurgitation: etiology, imaging findings, and preferred treatment strategies**. Caption, MR, mitral regurgitation; AF, atrial fibrillation; LA, left atrium; LV, left ventricle; MI, myocardial infarction; DCM, dilated cardiomyopathy; GDMT, guideline-directed medical therapy; CABG, coronary artery bypass grafting; MV, mitral valve; PCI, percutaneous coronary intervention; CRT, cardiac resynchronization therapy; TEER, transcatheter edge-to-edge repair; TMVI, transcatheter mitral valve implantation.

An important caveat to this three-category framework is that a clinically significant proportion of patients, estimated at approximately 20% in real-world populations, present a mixed phenotype, in which primary valvular involvement coexists with ventricular and/or atrial pathology [11,16]. In these patients, management is more complex and requires experienced multimodal evaluation to identify the predominant mechanism and guide the most appropriate treatment strategy. The etiology, characteristic imaging findings, and preferred treatment strategies for each MR category are summarized in Fig. 2.

Although mechanisms differ, the natural history of MR shares two recurring features. Significant MR, whatever its origin, accelerates atrial and ventricular remodeling, raises pulmonary pressures, and predisposes to arrhythmias. Moreover, the onset of HF symptoms typically marks an inflection point in prognosis, with a steep decline in survival unless the regurgitation is corrected [5]. For these reasons, contemporary MR management emphasizes early recognition and timely intervention, before irreversible damage occurs.

**Fig. 3.**
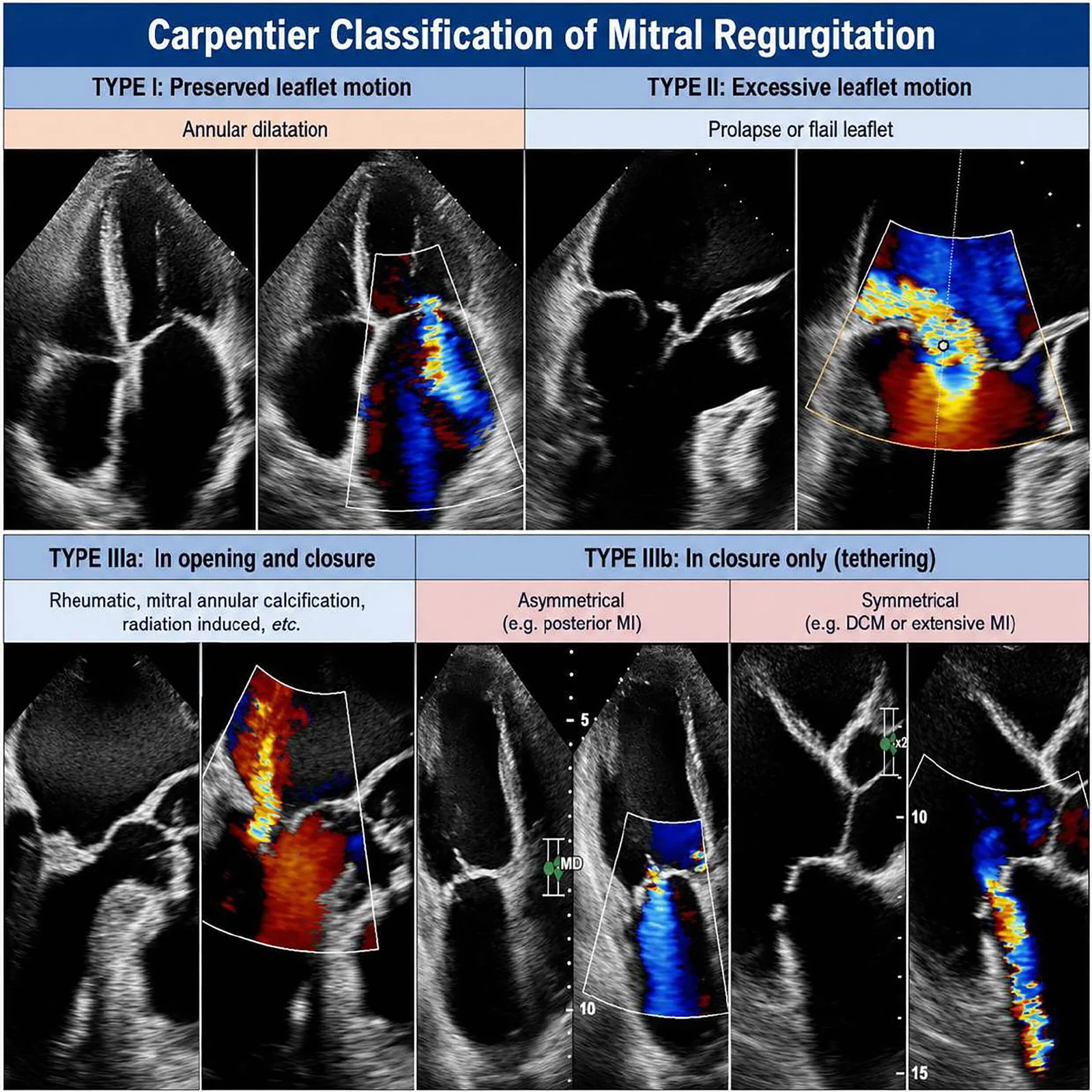
**Carpentier classification of mitral regurgitation**.

The relationship between the traditional Carpentier classification and the contemporary mechanism-based framework is illustrated in Fig. 1. While Carpentier’s anatomical classification (Fig. 3) remains fundamental for surgical planning by describing leaflet motion patterns, the mechanism-based approach provides prognostic stratification and guides selection of medical versus interventional therapy. Understanding both classification systems allows clinicians to integrate anatomical assessment with pathophysiological insight, optimizing individualized treatment strategies. Table 1 summarizes the key differences between the two systems.

**Table 1.** **Key differences between the Carpentier classification and the 2025 ESC/EACTS classification**.

Feature	Carpentier classification	2025 ESC/EACTS classification
Primary focus	Leaflet motion pattern	Underlying disease mechanism
Clinical utility	Surgical planning and repair feasibility	Prognosis and treatment selection
Treatment implications	Guides selection of surgical technique	Guides selection of medical therapy and/or interventional strategies

## 3. Diagnostic Approach

### 3.1 Echocardiography

Transthoracic echocardiography (TTE) represents the first-line imaging modality for assessing valve disease and chamber size and function. Standard TTE evaluation of the mitral valve includes two- and three-dimensional views, M-mode, and Doppler assessment. The constant spatial relationship of the MV with surrounding structures facilitates detailed anatomic evaluation. Transoesophageal echocardiography (TOE) serves as a second-level tool, providing superior spatial resolution due to its proximity to the left atrium. It is essential for detailed analysis of valve anatomy, MR grading, and for pre-procedural planning of surgical or transcatheter interventions. Three-dimensional echocardiography further enhances anatomical understanding by offering a surgical-like view of the MV and enabling multiplanar reconstructions that improve measurement accuracy and reproducibility.

**Fig. 4.**
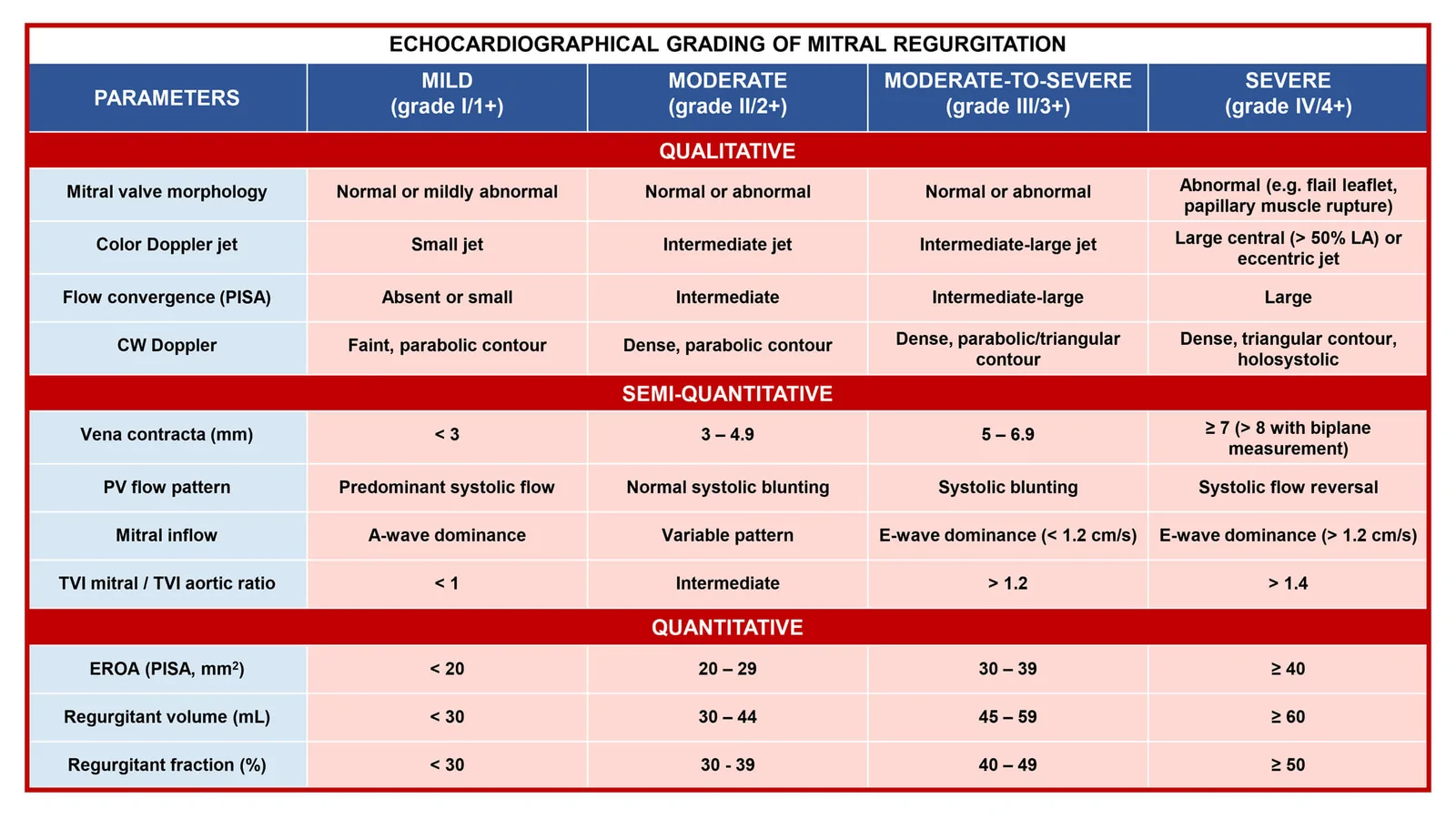
**Echocardiographic grading of mitral regurgitation (ASE/EACVI)**. EROA, effective regurgitant orifice area; CW, continuous-wave; TVI, time-velocity integral.

Assessment of Mitral Regurgitation Severity**. **Accurate echocardiographic grading of MR is crucial, as current guidelines recommend surgical intervention only for severe MR defined by standardized criteria [8,9]. Both European and American recommendations emphasize the need for an integrated approach combining qualitative, semiquantitative, and quantitative parameters. According to the American Society of Echocardiography (ASE) and the European Association of Cardiovascular Imaging (EACVI) recommendations, this approach should include several different echocardiographic criteria that allow MR to be classified as mild (Grade I/1+), moderate (Grade II/2+), moderate-to-severe (Grade III/3+), or severe (Grade IV/4+) [17,18,19]. A comprehensive summary of echocardiographic parameters by grade is provided in Fig. 4, incorporating qualitative, semiquantitative, and quantitative criteria aligned with ASE/EACVI guidance.

(1) Qualitative assessment primarily relies on color Doppler evaluation of the regurgitant jet. Larger and deeper jets generally indicate more severe MR, but jet size can be influenced by technical settings (aliasing velocity, gain) and hemodynamic conditions (blood pressure, jet direction). Eccentric jets, common in functional MR, may appear smaller due to the Coanda effect [20]. The density of the continuous-wave Doppler signal can also reflect MR severity, as higher signal intensity corresponds to larger regurgitant volumes. Finally, assessing the jet’s duration is essential: even with marked prolapse and a large regurgitant orifice, a brief telesystolic jet may result in a relatively low regurgitant volume.

(2) Semiquantitative approaches include measurements of (i) vena contracta (VC) width, (ii) pulmonary vein systolic flow, and (iii) mitral inflow velocity (E wave velocity). The VC width, measured in millimeters, represents the narrowest portion of the MR jet and corresponds to the diameter of the regurgitant orifice. A VC width ≥7 mm is generally indicative of severe MR [18]. However, because VC measurements are small (typically <1 cm), minor inaccuracies can result in significant errors in grading MR severity. In cases with elliptical or irregular orifices, single two-dimensional measurements may underestimate the lesion [21]. In such settings, three-dimensional vena contracta area (VCA) assessment provides a more reliable quantification by allowing direct measurement of the regurgitant orifice area. Pulmonary vein systolic flow, evaluated by pulsed-wave Doppler, provides indirect information about left atrial pressure and MR severity. In normal conditions, systolic flow exceeds diastolic flow (S wave taller than D wave). With moderate MR, the S wave becomes markedly blunted, while systolic flow reversal indicates severe MR or markedly elevated left atrial pressure. This method is not applicable in case of AF [18]. The mitral inflow pattern reflects the transmitral pressure gradient and is influenced by diastolic function. An elevated early filling velocity (E wave >1.2 cm/s), in the absence of mitral stenosis, supports the diagnosis of severe MR. Also, this method is not applicable in case of AF [18].

(3) Quantitative evaluation of MR relies on three main parameters: (i) effective regurgitant orifice area (EROA), i.e., the principal measure of lesion severity, representing the regurgitant orifice area; (ii) regurgitant volume (RgVol), i.e., the amount of blood regurgitated per systole, reflecting volume overload; (iii) regurgitant fraction (RF), i.e., the ratio of regurgitant to total stroke volume, providing a patient-specific index of severity. These parameters can be derived using three validated methods: the quantitative pulsed Doppler method, the quantitative volumetric method, and the flow convergence (PISA) method [22].

Quantitative Pulsed Doppler Method. By combining velocity-time integral (VTI) recordings with 2D or 3D measurements of flow area, stroke volumes can be calculated at different sites. In the absence of regurgitation, SVs through the mitral annulus, LV outflow tract (LVOT), and right ventricular outflow tract (RVOT) are equivalent. When a valve is regurgitant, the SV through that valve exceeds the forward SV of the non-regurgitant valves, and the difference represents the regurgitant volume. The regurgitant fraction is then obtained by dividing RgVol by the total SV through the regurgitant valve.

Quantitative Volumetric Method. Because blood is incompressible, the total LV stroke volume (SVLV) equals the sum of forward and mitral regurgitant volume. When the forward SV (measured through the non-regurgitant aortic valve) is known, RgVol can be derived as RgVol = SVLV – SVForward. Three-dimensional echocardiography improves accuracy by providing more reliable LV volume measurements.

Flow Convergence (PISA) Method. In valvular regurgitation, flow convergence occurs proximal to the orifice, forming hemispheric shells of increasing velocity. Color Doppler allows visualization of the aliasing hemisphere by shifting the baseline toward the regurgitant jet. The PISA radius (r) is measured from the aliasing boundary to the vena contracta, and EROA = (6.28 × r2 × Va) / PeakVRegJet, where Va is the aliasing velocity and PeakVRegJet is the peak velocity of the regurgitant jet by CW Doppler. Regurgitant volume is then calculated as RgVol = EROA × VTIRegJet (velocity time integral of the regurgitant jet velocity). Although the PISA method is widely used, it assumes a hemispheric flow geometry and a circular orifice. In functional MR with an elliptical or eccentric jet, these assumptions lead to underestimation [21]. Moreover, since PISA reflects instantaneous peak flow, the derived EROA may not correspond to the average regurgitant orifice throughout systole, particularly in late-systolic prolapse.

### 3.2 Cardiac Magnetic Resonance

CMR is widely regarded as the gold standard for the assessment of cardiac dimensions, function, and tissue characterization. It provides precise evaluation of myocardial scarring and viability, allowing accurate delineation of damaged versus healthy tissue. CMR has increasingly assumed an important role in the evaluation of MV disease, offering a reliable quantitative method to assess mitral regurgitation in cases where findings are inconclusive or ultrasound imaging is suboptimal. Its capability to measure the effective regurgitant volume without geometrical assumptions offers a significant advantage when evaluating complex or multiple regurgitant jets, multivalvular disease, or patients with poor echocardiographic windows. Although CMR demonstrates superior accuracy compared to echocardiography for quantifying MR severity in these challenging situations, the latter remains the preferred method for assessing the mechanism of regurgitation due to its higher spatial and temporal resolution. CMR achieves direct quantification of stroke volumes and regurgitant volumes through dedicated phase-contrast sequences, which measure the velocity of moving proton spins, essentially capturing blood flow. In recent years, a major advancement has emerged with the introduction of 4D flow imaging for valve and shunt evaluation. This technique acquires time-resolved, three-dimensional velocity data across a volume of interest, enabling retrospective flow analysis at any location within the acquired dataset without the need to predefine measurement planes. 4D flow captures velocity in all three spatial directions (x, y, z) throughout the cardiac cycle, providing a comprehensive, dynamic map of blood motion. Four primary methods are currently used to accurately quantify MR regurgitant volume with CMR [23,24]: (i) the difference between total LV stroke volume, calculated using planimetry from cine short-axis stacks, and aortic flow measured by 2D phase-contrast sequences; (ii) the difference between LV and right ventricular (RV) stroke volumes using planimetry of cine short-axis stacks, applicable in the absence of shunts or additional significant regurgitant lesions; (iii) the difference between total LV stroke volume and pulmonary flow measured by 2D phase-contrast sequences; (iv) direct quantification of MR flow using 4D flow CMR with mitral valve tracking.

MR Quantification with 2D Phase-Contrast Sequence. The 2D phase-contrast (PC) MRI sequence is specifically designed to measure and visualize blood flow velocity across a single imaging plane and constitutes the standard method for evaluating regurgitant valve lesions. This approach relies on the principle that moving spins, such as flowing blood, undergo a phase shift when traversing magnetic field gradients, with the magnitude of this shift being directly proportional to their velocity. To obtain accurate measurements of forward aortic flow, a through-plane imaging plane - perpendicular to the vessel - should be positioned at the sino-tubular junction. Mitral regurgitant volume is then calculated by subtracting the forward aortic stroke volume from the total LV stroke volume, which is obtained from a short-axis cine stack of the LV. Finally, the regurgitant fraction is determined as the ratio of regurgitant volume to total LV stroke volume [25].

Beyond regurgitation quantification, CMR offers a unique role in myocardial tissue characterization. The evaluation of chronic MR has traditionally relied on volumetric and functional parameters, primarily LV size and EF, which often fail to capture early myocardial injury, as they tend to remain within normal ranges until relatively advanced disease stages. In this context, tissue characterization techniques, particularly T1 mapping, have emerged as a powerful tool to detect subtle diffuse myocardial alterations that precede overt functional decline [26]. Unlike late gadolinium enhancement, which is primarily sensitive to focal replacement fibrosis, T1 mapping is uniquely suited to identify diffuse myocardial involvement. Kitkungvan et al. [27] reported that extracellular volume (ECV) quantified through T1 mapping was significantly associated with progression to MV surgery and allowed further risk stratification among patients with a regurgitant fraction ≥40%. These findings suggest that CMR tissue characterization may offer meaningful prognostic insight and assist in identifying patients at higher risk of unfavorable clinical evolution.

### 3.3 Cardiac Computed Tomography

CCT offers excellent spatial resolution and is highly reproducible, being relatively operator-independent. However, even with the most advanced scanners, which can achieve temporal resolutions of up to 66 ms, CCT temporal resolution remains inferior to that of echocardiography, and image quality is highly susceptible to artifacts related to arrhythmias. Nevertheless, cardiac CT provides comprehensive visualization of cardiac and vascular structures and can deliver detailed information regarding mitral annular shape and sizing, papillary muscle position and dimensions, LV shape and size, as well as the spatial relationship of the heart with the chest wall. AlthoughCT has a limited role in the assessment of MR severity, it plays a crucial role in pre-procedural planning for transcatheter interventions, including MV replacement, paravalvular leak closure, and transcatheter annuloplasty [28]. CT is the modality of choice for accurate prosthesis sizing and prediction of LVOT obstruction risk in the context of transcatheter mitral valve replacement (TMVR) planning, a critical step given that only a minority of patients screened for TMVR demonstrate anatomical suitability for the procedure.

In summary, multimodality imaging is essential not only for grading MR severity, but also for guiding the choice and timing of medical, surgical, or transcatheter treatment, as discussed in the following sections.

## 4. Management

MR management follows a stepwise approach aimed at preventing irreversible myocardial damage. Three therapeutic modalities are available, and their indication depends on MR mechanism, severity, and patient profile. Medical therapy plays a limited role in primary MR but is central to secondary MR, where GDMT and cardiac resynchronization therapy (CRT) are mandatory. Surgery remains the gold standard for primary MR when valve anatomy allows durable repair, with minimally invasive approaches now well-established as alternatives to conventional sternotomy. Among transcatheter options, M-TEER is recommended for patients at high or prohibitive surgical risk and is particularly important in ventricular secondary MR after GDMT optimization, while TMVR is emerging as an option for cases unsuitable for repair.

### 4.1 Medical Therapy

The role of medical therapy varies significantly across the spectrum of MR etiologies. In primary degenerative MR, pharmacological treatment has a very limited impact on the natural history. Vasodilators or diuretics can offer symptomatic relief in patients who await surgery or in those who are deemed inoperable, but they do not slow the progression of the disease. The only curative approach is mechanical correction of the valve, ideally by surgical repair. Patients with primary MR and impaired LV function should receive GDMT according to HF guidelines [29].

In contrast, in the presence of ventricular SMR, guidelines recommend that the therapeutic strategy be based initially on pharmacological therapy, namely on GDMT for HF [8,29]. Angiotensin receptor-neprilysin inhibitors (ARNi), beta-blockers, mineralocorticoid antagonists, and SGLT2 inhibitors have collectively reshaped outcomes in HFrEF, reducing LV volumes and afterload, and thereby often mitigating MR severity. Equally critical is CRT in eligible patients, since restoring interventricular synchrony improves papillary muscle position, augments leaflet coaptation and reduces regurgitant volume. Both clinical experience and trial designs have made it clear that interventional therapies for ventricular SMR provide the greatest benefit only after GDMT and CRT have been fully optimized [29,30,31].

In atrial SMR, the therapeutic foundation lies in the atrium itself: aggressive rhythm and rate control with pharmacological agents, catheter ablation of AF when feasible, and meticulous management of comorbidities such as hypertension, obesity, and sleep apnea reduce left atrial pressures, mitigate annular dilatation, and may partially reverse regurgitation [32].

**Fig. 5.**
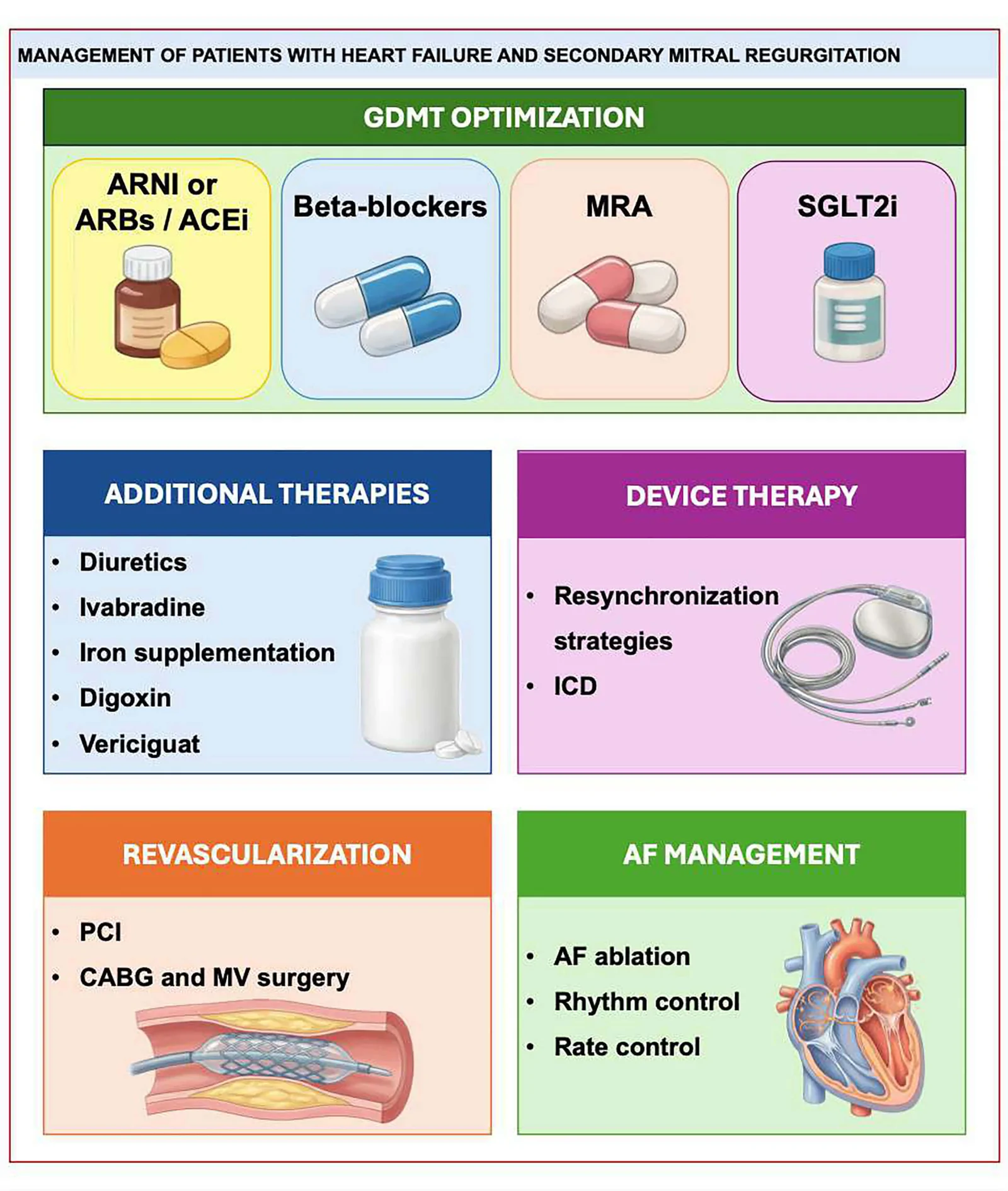
**Management of patients with HF and secondary MR**. Abbreviations: GDMT, guideline-directed medical therapy; ARNI, angiotensin receptor-neprilysin inhibitor; ARBs, angiotensin receptor blockers; ACEi, angiotensin-converting enzyme inhibitors; MRA, mineralocorticoid receptor antagonists; SGLT2i, sodium-glucose cotransporter-2 inhibitors; ICD, implantable cardioverter-defibrillator; PCI, percutaneous coronary intervention; CABG, coronary artery bypass grafting; MV, mitral valve; AF, atrial fibrillation; HF, heart failure.

A comprehensive summary of the management of patients with HF and secondary MR, including the four pillars of GDMT (ARNi/ARBs/ACEi, beta-blockers, MRA, SGLT2i), additional therapies (diuretics, ivabradine, iron supplementation, vericiguat, digoxin), device therapy (CRT, ICD), revascularization, and AF management, is provided in Fig. 5.

### 4.2 Surgery

Surgery has been the cornerstone of MR management for decades. Compared with conventional medical therapy, early surgery is associated with significantly improved outcomes in the long-term [33]. MV surgery encompasses operative techniques designed to correct structural or functional abnormalities of the MV, most commonly in the context of severe MR. The management of these patients should be discussed by a multidisciplinary HVT including HF specialists if necessary [8]. The decision between MV repair and replacement should be based on valve pathology, ventricular geometry, patient comorbidities, surgical risk, and local surgical expertise [8,9]. The goal is to provide a lasting correction of regurgitation while maintaining LV function.

**Fig. 6.**
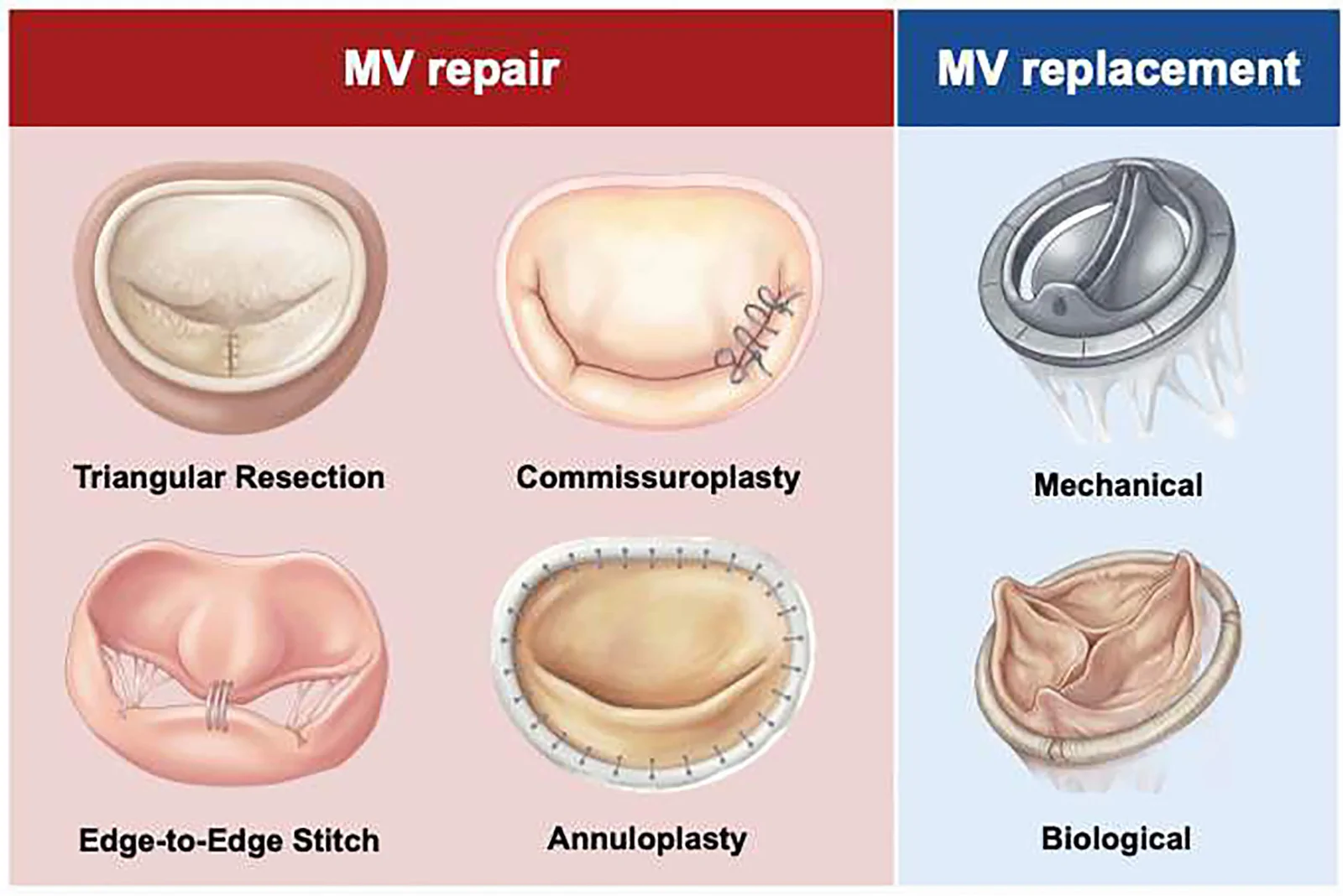
**Surgical techniques for mitral valve repair and replacement**. Caption: MV repair techniques include: triangular resection (excision of prolapsing/flail segment, typically posterior leaflet), edge-to-edge stitch (Alfieri technique creating double-orifice valve), commissuroplasty (repair of commissural prolapse or rheumatic fusion), and annuloplasty (ring insertion to restore annular geometry and enhance leaflet coaptation). When repair is not feasible, MV replacement is performed using either mechanical prostheses (durable but requiring lifelong anticoagulation) or biological prostheses (limited durability but no mandatory anticoagulation).

In primary MR, MV repair is recommended as the surgical technique of choice. It is generally preferred because it consistently yields superior outcomes compared with valve replacement, including lower peri-operative mortality (<1%), improved long-term survival and functional results, and a reduced incidence of valve-related complications when the anatomy is suitable and the expertise available [34,35]. MV repair allows for preservation of LV function and reduction of long-term mortality, and avoids the complications linked to prosthetic valves [36]. Accordingly, current guidelines recommend early referral to experienced centers for both symptomatic and asymptomatic patients, with surgery recommended for the latter category once markers of hemodynamic deterioration emerge (left ventricular ejection fraction (LVEF) ≤60%, LV end-systolic diameter ≥40 mm, AF, severe atrial enlargement, or pulmonary hypertension) [8]. The choice of repair technique depends on mechanism of regurgitation (Carpentier classification, Fig. 1), leaflet pathology, and surgeon expertise. Surgical MV repair typically involves resection of prolapsing or flail segments, implantation of artificial chordae, and edge-to-edge leaflet approximation (Fig. 6, Table 2). All repairs are typically reinforced by annuloplasty to restore annular geometry and ensure durability [37,38].

**Table 2.** **Surgical mitral valve repair techniques**.

Technique	Objective	Brief description	Main indications
Quadrangular or Triangular Resection	Eliminate prolapsing posterior leaflet tissue	Excision of a portion of the posterior leaflet (usually P2 segment) followed by leaflet reconstruction and annuloplasty	Degenerative MR with localized posterior leaflet prolapse or flail
Sliding Leaflet Plasty	Reduce posterior leaflet height after resection	Posterior leaflet detachment and downward reattachment to annulus to prevent SAM	Posterior leaflet redundancy with excess height or large resections
Neochordae (PTFE Chordal Replacement)	Restore leaflet coaptation	Implantation of artificial PTFE chords anchored to papillary muscles	Anterior or bileaflet prolapse, flail chordae, ruptured primary chords
Chordal Transfer/Transposition	Redistribute native chordal support	Transfer of intact secondary chordae to the prolapsing leaflet	Isolated posterior or anterior leaflet prolapse with intact secondary chords
Annuloplasty	Restore annular geometry and leaflet coaptation	Implantation of a prosthetic ring or band (rigid, semi-rigid, or flexible)	All degenerative or functional MR repairs; essential for durability
Edge-to-Edge (Alfieri) Stitch	Create a double-orifice valve to improve coaptation	Central suturing of A2 and P2 scallops to eliminate central regurgitation jet	Central jet MR, limited prolapse, or adjunct to other repair techniques
Commissuroplasty	Restore commissural alignment	Plication or suture of commissural areas	Commissural MR or localized prolapse near commissures
Papillary Muscle Relocation or Approximation	Reduce tethering and restore coaptation in functional MR	Repositioning or approximation of papillary muscles closer to annulus	Ischemic or functional MR with significant leaflet tethering
Secondary Chordal Cutting	Improve leaflet mobility	Selective transection of restrictive secondary chords	Functional/ischemic MR with restricted posterior leaflet motion
Leaflet Augmentation (Patch Repair)	Increase leaflet surface area	Pericardial patch augmentation of restricted leaflet	Rheumatic or ischemic MR with retracted leaflets or inadequate coaptation surface

In case of isolated posterior leaflet prolapse, especially in the P2 segment, repair success rates exceed 95% at experienced centers. In these situations, replacement should only be considered if repair has been attempted and failed. Instead, anterior leaflet or bileaflet disease requires more complex repairs, such as artificial chordae, extensive leaflet reconstruction or combined approaches, and should be referred to experienced mitral surgeons at high-volume centers [39,40]. Nonetheless, repair remains preferable to replacement whenever anatomically feasible [8,9]. MV replacement is reserved for cases where repair is not feasible or is unlikely to provide durable results, such as extensive leaflet destruction, severe calcification, rheumatic involvement, or advanced ischemic secondary MR. Replacement can be performed using mechanical or bioprosthetic prostheses, selected according to patient characteristics and expected durability requirements (Table 3). Mechanical prostheses offer long-term durability but require lifelong anticoagulation due to thromboembolic risk. Bioprosthetic valves have lower thromboembolic risk but limited durability, making them preferable in older patients or in those with contraindications to life-long anticoagulation [41,42]. Preservation of the subvalvular apparatus during replacement is critical to maintaining LV function.

**Table 3.** **Mitral valve prostheses**.

Mechanical	Biological (Porcine)	Biological (Pericardial)
Starr-Edwards (caged ball)	Hancock I	Carpentier-Edwards Perimount
Tilting disk (Medtronic Hall, Omnicarbon)	Hancock II	
Bileaflet (St. Jude Medical, Carbomedics, ATS, On-X)	Carpentier-Edwards Porcine	
	Mosaic	
	St. Jude Biocor	

When considering secondary MR, surgery should follow GDMT and device therapy when indicated. Randomized trials and observational studies have not demonstrated a survival benefit of isolated mitral surgery over medical therapy or transcatheter interventions [43]. Surgical options are mainly reserved for patients undergoing other cardiac surgeries or for those with anatomy unsuitable for transcatheter therapies. Restrictive annuloplasty can reduce regurgitation in the short term but is associated with a high recurrence rate, particularly with advanced ventricular remodelling or ischemic disease. In severe ischemic SMR, chordal-sparing MV replacement may be preferred over downsized annuloplasty [43]. The MATTERHORN trial recently demonstrated that M-TEER resulted in non-inferior 1-year outcomes compared with surgical mitral valve repair/replacement in secondary MR, with lower procedural risk, further supporting transcatheter approaches as first-line interventional therapy in eligible SMR patients [44]. In atrial SMR, surgical annuloplasty combined with AF ablation with the Maze procedure (when indicated) has been shown to improve symptoms and outcomes [45,46].

### 4.3 Surgical Approach: Minimally Invasive vs Sternotomy

The choice of surgical access should be tailored to each individual patient, as it may significantly impact perioperative outcomes and recovery. Minimally invasive mitral valve surgery (MiMVS), including right minithoracotomy, robotic-assisted procedures, and transapical beating-heart neochordal implantation, is recommended in appropriately selected patients at experienced centers. Both observational and randomized studies have shown that MiMVS achieves comparable efficacy and durability to conventional sternotomy while simultaneously offering meaningful advantages, including reduced surgical trauma, blood loss, lower rates of postoperative AF, shorter hospital stay, recovery time, and superior cosmetic results [47,48]. Long-term data from a 21-year single-center experience demonstrate similar all-cause mortality and freedom from MV-related reoperation between MiMVS and conventional sternotomy [49]. Nevertheless, median sternotomy remains the preferred approach for patients requiring multiple concurrent procedures (e.g., multivalve surgery, coronary revascularization) or complex procedures (e.g., uncertain valve exposure, redo cardiac surgery, emergency surgery). When selecting the optimal surgical approach, the HVT should always consider anatomical complexity, procedural risk, and institutional expertise. Robot-assisted techniques are applicable to a broad spectrum of mitral pathologies, including degenerative, rheumatic, and functional MR, and can be safely performed even on elderly patients or complex cases in experienced centers [50,51]. Limitations include high procedural costs, the requirement of specialized infrastructure and team training, and a steep learning curve. In summary, median, or conventional, sternotomy is the most frequently utilized approach for MV surgery, as it is carried out routinely in most hospitals. MiMVS has recently established itself as a safe and effective alternative to conventional surgery, offering excellent outcomes and enhanced patient recovery when performed by experienced teams. The main drawback is their limited availability across national healthcare systems. Robot-assisted techniques are still a prerogative of the most advanced Heart Valve Centers.

### 4.4 Transcatheter Therapies

Over the last decade, transcatheter interventions have substantially changed how MR is treated across its different phenotypes, and are now part of the therapeutic algorithm in the 2025 ESC/EACTS guidelines. The indication for a transcatheter rather than surgical approach differs substantially between primary and secondary MR [8].

Transcatheter edge-to-edge repair (TEER) is an evolution of the surgical edge-to-edge repair, first performed by Professor Ottavio Alfieri in 1991 [52,53]. The earliest evidence on TEER emerged in the context of primary degenerative MR, particularly in patients at prohibitive surgical risk. The EVEREST II trial established the safety and feasibility of the MitraClip device, demonstrating significant symptom improvement and functional benefit compared with medical therapy. However, crossover to surgery was frequent, and residual ≥2+ MR was substantially higher with TEER compared with surgical repair (57% vs. 24%), underlining that surgery remains the gold standard in anatomically repairable valves with acceptable risk profiles [54]. This also highlights the complexity of patient selection in the context of primary MR, with the goal being to recommend TEER only in those patients who present an ideal MV anatomy. The anatomical features more amenable to this technique include a central A2/P2 prolapse or flail with flail gap <10 mm, flail width <15 mm, mitral valve area >4 cm^2^, and transmitral gradient <4 mmHg. Complex Barlow’s disease, commissural prolapse, severe annular or leaflet calcification, or multi-segment pathology reduces procedural success and durability, requiring evaluation by HVT in specialized centers. The CLASP IID randomized trial studied a total of 300 patients with symptomatic severe degenerative MR and a prohibitive surgical risk. It demonstrated that the PASCAL system is non-inferior to MitraClip in prohibitive-risk primary MR, with similar improvements in symptoms and quality of life [55]. For primary MR, surgery (preferably repair) remains the standard of care for patients at low or intermediate surgical risk. The 2025 ESC/EACTS guidelines provide a Class IIa, Level B recommendation for M-TEER when the patient is at high surgical risk and the anatomy is suitable as assessed by the HVT [8]. TEER has made a far more transformative impact in patients with ventricular secondary MR. Both observational and randomized studies elevate it as a potential disease-modifying therapy, while simultaneously defining its limitations. Table 4 summarizes the three landmark randomized controlled trials (RCTs): COAPT, MITRA-FR, and RESHAPE-HF2. The COAPT trial selected patients with severe MR and HFrEF still symptomatic despite optimized GDMT, including CRT when indicated, and with anatomic features amenable to clip placement (COAPT criteria, details below). Compared to medical therapy alone, TEER reduced all-cause mortality by about 40% and halved HF hospitalizations at 2 years [56], with durable benefits at 5 years [57]. This trial cemented TEER as a therapy that alters prognosis when MR is a dominant driver of symptoms. The MITRA-FR trial enrolled patients with severe MR and HFrEF with markedly dilated left ventricles. In this trial, M-TEER did not improve survival and did not reduce HF hospitalizations compared to GDMT [58]. This study underscored that MR correction is futile when regurgitation is merely a bystander of advanced cardiomyopathy. The apparent discordance between these studies is now understood as a function of patient selection and proportionality: when MR is disproportionately severe relative to LV size and is a dominant driver of symptoms, its correction improves prognosis; when MR is simply the epiphenomenon of advanced cardiomyopathy, correction does not improve the patient’s outcome. The RESHAPE-HF2 trial recently reinforced this principle. It studied a cohort of 505 patients with severe ventricular SMR and HFrEF, meticulously selected on anatomical characteristics and clinical history. Compared to GDMT alone, M-TEER significantly reduced the composite outcome of recurrent HF hospitalization events and cardiovascular death, while also registering an improvement on quality-of-life scales [59]. These three RCTs were recently analyzed in a study-level meta-analysis, which demonstrated a clear-cut benefit of M-TEER in terms of HF hospitalizations and survival rates, although the latter did not reach statistical significance [60]. A retrospective analysis of the prospective EuroSMR Registry found that, in a population of individuals with secondary MR and HFrEF, M-TEER allowed up-titration of GDMT at follow-up in a considerable proportion of patients, a feature independently associated with lower rates of mortality and HF hospitalizations [61]. Based on this evidence, the 2025 ESC Guidelines now recommend M-TEER (Class I, Level B) in hemodynamically stable, symptomatic patients with LVEF <50% and persistent severe ventricular SMR despite optimized GDMT and CRT (if indicated), fulfilling specific clinical and echocardiographic criteria (Table 5) [8].

**Table 4.** **Landmark RCTs on M-TEER in ventricular secondary MR**.

Feature	COAPT (2018)	MITRA-FR (2018)	RESHAPE-HF2 (2024)
Patients enrolled	614	307	505
Study arms	M-TEER + GDMT vs GDMT	M-TEER + GDMT vs GDMT	M-TEER + GDMT vs GDMT
Baseline LVEF (%)	31.3 ± 9.1	33.3 ± 6.5	32 (26–37)
Baseline LVEDV (ml)	136 ± 37	253 ± 69	200 (153–249)
Baseline EROA (cm^2^)	0.41 ± 0.15	0.31 ± 0.1	0.23 (0.20–0.30)
Primary endpoint	Annualized HF hospitalizations + safety at 12 months	Death/HF hospitalization at 12 months	Composite HF hospitalization/CV death at 24 months + KCCQ at 12 months
Result	POSITIVE: HF hosp 35.8% vs 67.9% (HR 0.53; *p* < 0.001)	NEUTRAL: 54.6% vs 51.3% (OR 1.16; *p* = 0.53)	POSITIVE: 37.0 vs 58.9 events/100 pt-yrs (*p* = 0.002)

**Key Points**: COAPT demonstrated benefit in disproportionate severe SMR (MR dominant driver, relatively preserved LV). MITRA-FR was neutral in proportionate SMR (markedly dilated LV with modest MR, cardiomyopathy was the main driver). RESHAPE-HF2 confirmed efficacy even in moderate-to-severe SMR. The common principle: M-TEER benefits patients in whom MR is a dominant driver of symptoms, not merely an epiphenomenon of advanced cardiomyopathy. SMR, secondary MR; LVEF, left ventricular ejection fraction; LVEDV, left ventricular end-diastolic volume; CV, cardiovascular; KCCQ, Kansas City cardiomyopathy questionnaire; M-TEER, mitral transcatheter edge-to-edge repair.

**Table 5.** **Favorable and unfavorable features for M-TEER in secondary mitral regurgitation**.

Favorable features for TEER	Unfavorable features for TEER
Severe symptomatic MR disproportionate to LV dilation	MR proportionate to severe LV dilation (very large LV volumes with modest MR)
LVEF typically >20–25% and LV end-systolic dimension <70 mm (not end-stage)	Severely reduced LVEF (<20%) or markedly dilated LV (LVESD ≥70 mm)
Optimized GDMT and CRT already instituted, with persistent severe MR	Lack of GDMT/CRT optimization prior to intervention
Suitable valve anatomy: central regurgitant jet, adequate leaflet tissue for grasping, coaptation gap <10 mm, coaptation length ≥2 mm, mitral valve area >4 cm^2^, mean transmitral gradient <4 mmHg	Anatomic limitations: large flail gap (>10 mm), short coaptation length, severe leaflet/annular calcification, commissural jets, or complex Barlow’s anatomy
Absence of prohibitive annular or leaflet calcification	Severe right ventricular dysfunction or severe pulmonary hypertension
Patients not in advanced/end-stage HF where LV assist device or transplant would be more appropriate	

LVESD, left ventricular end-systolic diameter.

When anatomy is unsuitable for TEER, such as with large coaptation gaps, extensive calcification, or complex multi-jet lesions, TMVR has emerged as an alternative [62,63,64]. Several modern devices have shown near-complete elimination of MR and sustained efficacy at follow-up in early feasibility studies. However, procedural complexity, the risk of LVOT obstruction, and the non-negligible early mortality observed in fragile patients should not be underestimated. At present, TMVR is therefore reserved to expert centers and clinical trials, but it remains an important option for patients with otherwise limited treatment alternatives.

**Fig. 7.**
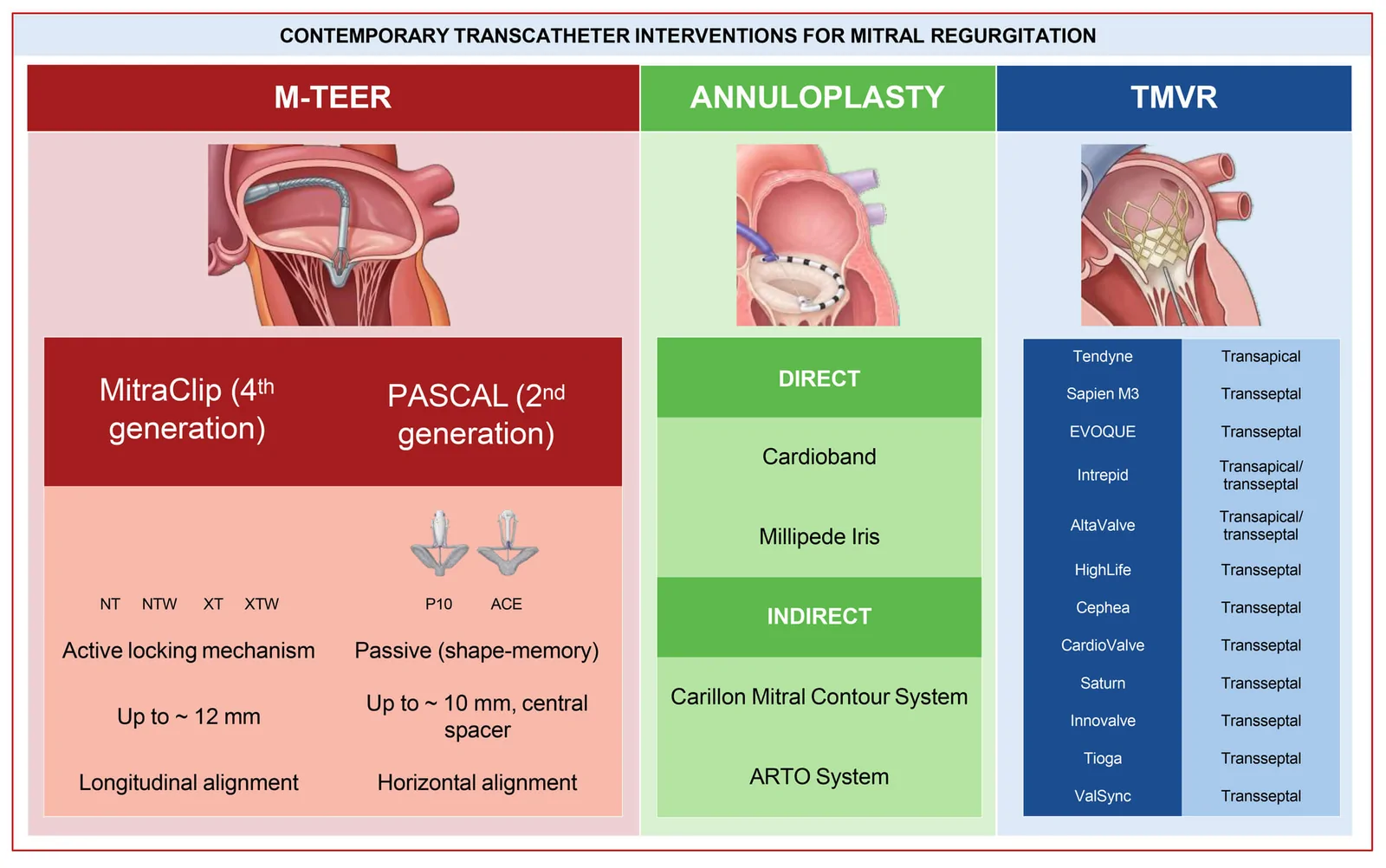
**Contemporary transcatheter interventions for mitral regurgitation**. TMVR, transcatheter mitral valve replacement.

Evidence for atrial MR remains less definitive, but the 2025 ESC/EACTS guidelines provide a Class IIb, Level B recommendation for M-TEER in patients with atrial SMR who remain symptomatic despite GDMT and are deemed at high surgical risk by the HVT [8,65]. Treatment of atrial SMR mainly relies on rhythm control, optimal management of comorbidities, and reduction of atrial pressure and volume overload through medical therapy. As ongoing prospective studies progress, the therapeutic algorithm for atrial SMR will likely become more clearly defined. Fig. 7 provides a landscape of the available devices for M-TEER, transcatheter annuloplasty, and TMVR.

## 5. Integrated Management Strategy: A Stepwise Approach

**Fig. 8.**
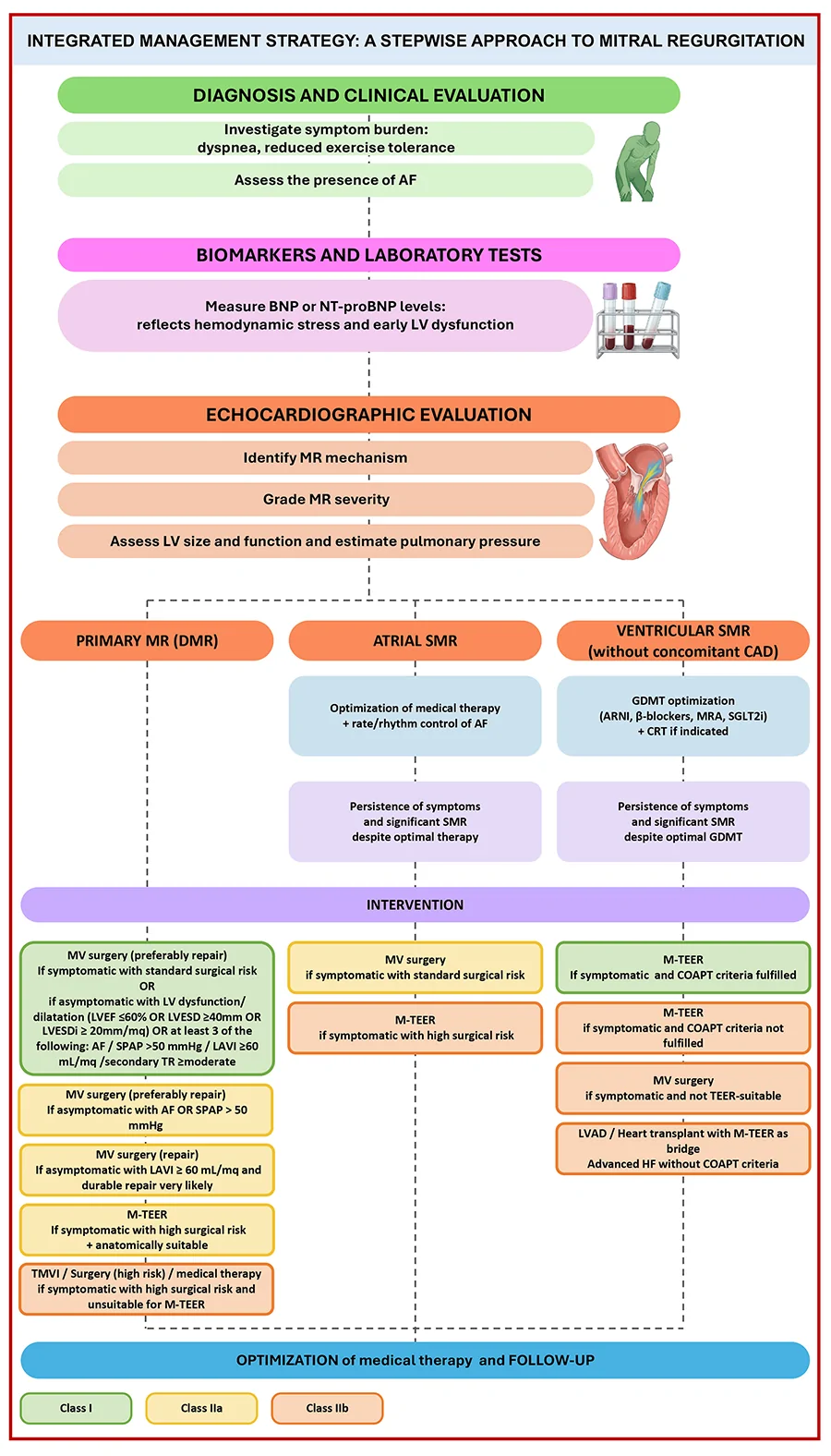
**Integrated management strategy: a stepwise approach**. Abbreviations: AF, atrial fibrillation; ARNI, angiotensin receptor–neprilysin inhibitor; ASMR, atrial secondary mitral regurgitation; BNP, B-type natriuretic peptide; CAD, coronary artery disease; COAPT, Cardiovascular Outcomes Assessment of the MitraClip Percutaneous Therapy for Heart Failure Patients with Functional Mitral Regurgitation; CRT, cardiac resynchronization therapy; DMR, degenerative mitral regurgitation; GDMT, guideline-directed medical therapy; HF, heart failure; LA, left atrial; LAVI, left atrial volume index; LV, left ventricular; LVAD, left ventricular assist device; LVEF, left ventricular ejection fraction; LVESD, left ventricular end-systolic diameter; LVESDi, left ventricular end-systolic diameter indexed to body surface area; M-TEER, mitral transcatheter edge-to-edge repair; MR, mitral regurgitation; MRA, mineralocorticoid receptor antagonist; MV, mitral valve; NT-proBNP, N-terminal pro–B-type natriuretic peptide; PMR, primary mitral regurgitation; SGLT2i, sodium–glucose cotransporter 2 inhibitor; SMR, secondary mitral regurgitation; SPAP, systolic pulmonary artery pressure; TMVI, transcatheter mitral valve implantation; TR, tricuspid regurgitation; VSMR, ventricular secondary mitral regurgitation.

The stepwise management of MR can be organized around three key decision points. The first is the mechanism of disease (primary, ventricular secondary, or atrial secondary), which directly shapes treatment decisions. The second is symptom status, together with markers of hemodynamic deterioration such as LV dysfunction, AF, pulmonary hypertension, and rising natriuretic peptides, all of which help identify patients who may benefit from early intervention. The third is surgical risk and anatomical suitability for repair, replacement, or transcatheter intervention. A clinical decision-making flowchart summarizing this framework is provided in Fig. 8.

Clinical and echocardiographic assessment. Management begins with a clinical and echocardiographic evaluation designed to define MR etiology and stratify the risk of disease progression. The following elements should be systematically assessed: MR mechanism (primary/degenerative or secondary/functional); severity of regurgitation, based on an integrated multiparametric imaging approach; LV size, systolic function (left ventricular end-systolic diameter (LVESD) >40 mm or LVEF <60%) and pulmonary pressure; symptom burden, including exertional dyspnea or reduced exercise tolerance; measurement of natriuretic peptides (B-type natriuretic peptide (BNP) or N-terminal pro–B-type natriuretic peptide (NT-proBNP)); presence of AF. In patients with non-severe MR, a strategy of watchful waiting supported by periodic clinical and echocardiographic evaluation is appropriate: mild MR every 2–3 years; moderate MR every 1–2 years. Follow-up intervals may be shortened in the presence of evolving symptoms, new-onset AF, pulmonary hypertension, LV remodeling, or rising natriuretic peptide levels. Medical therapy should be individualized according to MR etiology and patient characteristics. While medical therapy does not alter the natural history of primary MR, it plays a central role in secondary MR, where optimization of GDMT for HF is mandatory prior to considering any intervention.

Timing of intervention: the role of markers of disease progression. An adequately executed follow-up regimen must consider not only the clinical evolution of the patient’s symptoms but also analyze specific markers that are known to be associated with disease progression. The goal is to provide personalized risk stratification in order to identify the optimal timing for intervention.

LV remodeling. Traditional thresholds (LVEF ≤60% or LVESD ≥40 mm) may detect LV dysfunction too late, as progressive LV enlargement and early reduction in longitudinal strain may precede these classical cut-offs [66]. These parameters should therefore be integrated with serial biomarker assessment and symptom re-evaluation [67].

Pulmonary hypertension. Increased pulmonary pressures are correlated with worse outcomes in patients with MR. The 2025 ESC/EACTS guidelines provide a Class IIa recommendation for surgery in asymptomatic severe primary MR with systolic pulmonary artery pressure (SPAP) >50 mmHg. Patients with severe pulmonary hypertension (SPAP ≥70 mmHg) are unlikely to have improved outcomes even after intervention. Even modest increases in pulmonary pressure during routine follow-up should trigger reassessment at a Heart Valve Center [68].

Atrial fibrillation. New-onset or paroxysmal AF in a patient with moderate-to-severe MR is associated with significantly worse long-term outcomes compared to sinus rhythm, and should prompt early referral for intervention assessment [69,70]. When MV surgery is performed, concomitant surgical AF ablation is recommended if necessary [71].

Natriuretic peptides. BNP and NT-proBNP serve as quantitative markers of myocardial hemodynamic stress that can identify high-risk patients before overt symptomatic LV dysfunction develops. Elevated or progressively rising levels, even in asymptomatic patients, may prompt earlier referral to specialized centers before traditional intervention triggers appear, and serve as useful markers for post-procedural follow-up [72,73].

Frailty. Assessment of frailty has become integral to multidisciplinary team evaluation, particularly for elderly patients and those with severe comorbidities. The decision between conservative management and intervention must account not only for the cardiovascular pathology but also for the patient’s functional status, life expectancy, and personal preferences. A careful assessment of the risks and potential benefits of surgical or transcatheter procedures, including the possibility of procedural failure or perioperative death, which are higher in frail individuals, is essential [74,75]. The presence of any of these adverse features should prompt consideration for earlier intervention rather than continued surveillance.

Selection of interventional strategy. The choice of interventional strategy should be tailored to the individual patient and depends on disease etiology, valve anatomy, surgical risk, frailty, and institutional expertise. Surgical MV repair remains the preferred option for primary MR, given its better outcomes and durability when the anatomy is suitable and the procedure is performed by an experienced surgical team. Surgical MV replacement is indicated when durable repair is unlikely, for example in the presence of severe leaflet or annular calcification, or of complex degenerative anatomy. M-TEER is the preferred option for patients with primary or secondary MR at high or prohibitive surgical risk. In ventricular SMR, M-TEER is considered only when the patient is still symptomatic, or shows signs of hemodynamic deterioration, despite maximal GDMT up-titration. Newer TMVR devices have shown promising results in early registries but have not yet been incorporated into the guidelines.

Post-Intervention Management. Following surgical repair or replacement or TEER or TMVR, a structured program of post-procedural visits is crucial. Generally, an early echocardiographic and clinical evaluation is scheduled in the first 2–3 months after the procedure, followed by regular follow-up visits every 6–12 months. Continued management of the patient’s comorbidities is also paramount, including continuation and/or up-titration of HF medical therapy (particularly in ventricular SMR), management or prevention of AF and other arrhythmias, and endocarditis prophylaxis when indicated. Natriuretic peptides may also serve as adjunctive markers to assess post-operative hemodynamic improvement and detect early recurrence of dysfunction.

Special Scenarios. When MR is present in patients with hemodynamically significant coronary artery disease, the preferred strategy of treatment includes concomitant surgical coronary revascularization and MV surgery [76]. This procedure is recommended in patients with severe MR with suitable anatomy and may be considered when MR is moderate [77]. In patients deemed unfit for surgery, percutaneous revascularization with percutaneous coronary intervention (PCI) may be considered as the first step, followed by a re-evaluation of MR severity and symptoms, and potentially by M-TEER [78]. Acute MR complicating myocardial infarction, typically caused by papillary muscle rupture, represents a hemodynamic emergency requiring immediate stabilization with vasodilators, diuretics, and mechanical circulatory support (intra-aortic balloon pump or other devices), followed by urgent surgical intervention [79,80]. Transcatheter approaches may serve as a bridge in hemodynamically unstable patients at prohibitive immediate surgical risk, though outcomes are inferior to surgery in this acute setting [81]. Concomitant MR and tricuspid regurgitation (TR): functional TR frequently accompanies MR due to right heart pressure overload and annular dilatation. Current guidelines recommend concomitant tricuspid annuloplasty at the time of left-sided valve surgery when TR is at least moderate or tricuspid annular diameter exceeds 40 mm, even when TR is not hemodynamically significant, to prevent late right HF. When MR is treated percutaneously, transcatheter tricuspid intervention may be considered in selected patients with persistent significant TR [8].

## 6. Heart Valve Team

The management of MR has progressively moved towards a multidisciplinary approach built around the Heart Valve Team, which brings together clinical, imaging, surgical, and interventional expertise [82]. The HVT should include: a general cardiologist or HF specialist who provides longitudinal patient care and medical therapy optimization; a cardiologist with imaging expertise (echocardiography, CMR, CT) responsible for mechanistic diagnosis and severity grading; an interventional cardiologist with expertise in transcatheter mitral procedures; a cardiac surgeon with experience in mitral valve repair and replacement; other specialists (nephrologist, pulmonologist, geriatrician, palliative care physician) as indicated by the patient’s comorbidity profile. Each member contributes a specific perspective: the clinical cardiologist focuses on symptom burden and risk trajectory, the imaging specialist on anatomy and hemodynamics, the interventional cardiologist on transcatheter feasibility, and the surgeon on the likelihood of repair and operative risk. Given the heterogeneity of MR mechanisms and patient profiles, no single specialist can optimally define timing or modality of intervention. As recommended by current guidelines, the HVT provides a structured framework in which diagnosis, treatment, and follow-up are coordinated within experienced centers. Beyond procedural selection, the HVT helps to integrate multimodality imaging, evaluates the interplay between valve pathology, ventricular and atrial function, and comorbidities, and selects the most appropriate therapeutic strategy. This collaborative approach allows intervention to be carried out at the optimal time, before irreversible ventricular or atrial remodeling occurs [83]. Shared decision-making is an essential component of this approach, combining clinical judgement with patient preferences and personal goals. Transparent discussion of procedural risks, expected outcomes, and quality-of-life implications is particularly important in elderly or comorbid patients. The HVT supports this dialogue and ensures that treatment choices take into account both available evidence and patient values, including the option of conservative or palliative management when an invasive procedure is unlikely to provide a meaningful benefit [84]. Patient selection and procedural planning are the operational core of the HVT. Detailed pre-procedural imaging and collaborative planning ensure technical feasibility and safety, while coordinated follow-up verifies the durability of results and guides further care. The HVT is the operative mechanism through which the stepwise management algorithm described in Section 5 is translated into individualized clinical decisions. By combining expertise with patient-centered principles, the HVT translates complex therapeutic choices into tailored pathways, ultimately improving outcomes and quality of life in patients with MR.

## 7. Conclusion

MR is a heterogeneous and progressive disease in which early recognition and timely intervention are essential to prevent irreversible cardiac damage. Multimodality imaging, modern surgical techniques, and the expansion of transcatheter therapies have broadened treatment options across all MR phenotypes, including patients previously considered unsuitable for intervention. Effective management depends increasingly on dedicated HVTs, which ensure appropriate patient selection, coordinated decisions, and individualized treatment pathways. The ultimate goal is to modify the natural history of MR by intervening before advanced remodeling develops, improving survival, symptoms, and quality of life.

Several developments are likely to influence MR management in the coming years. In diagnostics, the integration of artificial intelligence-assisted echocardiographic analysis and CMR-based myocardial fibrosis quantification may enable earlier and more precise identification of patients at risk of irreversible remodeling [85]. In therapeutics, next-generation TMVR devices, currently demonstrating encouraging early outcomes in registries, are likely to gain guideline endorsement once longer-term data mature, potentially offering a new treatment pathway for patients with complex anatomy unsuitable for repair or TEER [86]. Dedicated prospective trials for atrial SMR will clarify the role of transcatheter annuloplasty and TEER in this growing population. Finally, novel biomarkers and wearable monitoring devices may allow dynamic, real-time risk stratification, moving beyond the current static assessment model.
